# Direct medical costs of end-stage kidney disease and renal replacement therapy: a cohort study in Guangzhou City, southern China

**DOI:** 10.1186/s12913-020-4960-x

**Published:** 2020-02-14

**Authors:** Hui Zhang, Chao Zhang, Sufen Zhu, Hongjian Ye, Donglan Zhang

**Affiliations:** 1 School of Public Health, Sun Yat-sen University, No. 74, Zhongshan Road 2, Guangzhou, China; 2 Business School, Sun Yat-sen University, No. 135, Xingang Xi Road, Guangzhou, China; 3 Department of Nephrology, The First Affiliated Hospital, Sun Yat-sen University, 58th, Zhongshan Road II, Guangzhou, 510080 China; 4 Department of Health Policy and Management, College of Public Health, University of Georgia, 100 Foster Road, Wright Hall 205D, Athens, GA 30602 USA

**Keywords:** End-stage kidney disease, Cost, Renal replacement therapy, Health policy, China, Health insurance

## Abstract

**Background:**

Renal replacement therapy was a lifesaving yet high-cost treatment for people with end-stage kidney disease (ESKD). This study aimed to estimate the direct medical costs per capita of ESKD by different treatment strategies: haemodialysis (HD); peritoneal dialysis (PD); kidney transplantation (KT) (in the first year); KT (in the second year), and by two urban health insurance schemes.

**Methods:**

This was a retrospective observational cohort study. Data were obtained from outpatient and inpatient claims database of two urban health insurance from Guangzhou City, Southern China. Adult patients with HD (*n* = 3765; mean age 58 years), PD (*n* = 1237; 51 years), KT (first year) (*n* = 117; 37 years) and KT (second year) (*n* = 41; 39 years) were identified between 2010 and 2012. The primary outcome was the annual per patient medical costs in 2013 Chinese Yuan (CNY) incurred in the outpatient and inpatient sectors. Secondary outcomes were annual outpatient visits and inpatient admissions, length of stay per admission. Generalized linear regression and bootstrapping statistical methods were used for analysis.

**Results:**

The estimated average annual medical costs for patients on HD were CNY 94,760.5 (US$15,066.0), 95% Confidence Interval (CI): CNY85,166.6–106,972.2, which was higher than those for patients on PD [CNY80,762.9 (US$12,840.5), 95% CI: CNY 76,249.8-85,498.9]. The estimated annual cost ratio of HD versus PD was 1.17 (95% CI: 1.12–1.25). Among the transplanted patients, the estimated average annual medical costs in the first year were CNY132,253.0 (US$21,026.9), 95%CI: CNY114,009.9–153,858.6, and in the second year were CNY93,155.3 (US$14,810.8), 95%CI: CNY61,120.6–101,989.1. The mean annual medical costs for dialysis patients under Urban Employee-based Basic Medical Insurance scheme were significantly higher than those for patients under Urban Resident-based Basic Medical Insurance scheme (*P* < 0.001).

**Conclusions:**

The direct medical costs of ESKD patients were high and different by types of renal replacement therapy and insurance. The findings can be used to conduct cost-effectiveness research on different types of RRT for ESKD patients that provides economic evidence for health policy design in China.

## Background

End-stage kidney disease (ESKD) was a leading cause of morbidity and mortality worldwide [1]. Renal replacement therapy (RRT), through either dialysis or kidney transplantation (KT), was a lifesaving yet high-cost treatment for people with ESKD [2]. Globally, the number of people receiving RRT was projected to be around 5.439 million by 2030, and the largest absolute growth in the number of people receiving RRT was in Asia, rising from 0.968 million people in 2010 to a projected 2.162 million people by 2030 [2]. In China, the prevalence of patients with ESKD on maintenance haemodialysis (HD) or peritoneal dialysis (PD) was 71.9 per one million population in 2008 [3]. The annual incidence of ESKD patients in mainland China was 36.1 per one million population in 2008 [3]. The prevalence of dialysis was lower in China than in many developed countries, and this reflected the unmet need for ESKD therapy due to a lack of financial and clinical resources of many Chinese patients [4].

The provision of RRT for patients with ESKD imposed a heavy financial burden on the health care systems in many countries [5]. It was estimated that over 1 trillion dollars were spent on ESKD globally [6]. In China, the total costs associated with ESKD were forecasted to be Chinese Yuan (CNY) 600.3 million (US$92.4 million) by 2025 [7]. The main challenges to expand the dialysis treatment included the high out-of-pocket (OOP) expenses and the growing inequalities in access to health care across different socioeconomic groups [8]. These issues were the main targets of China’s recent health care reform [9]. This reform was designed to enhance financial protection by covering all the urban residents with one of the basic insurance schemes, which included the Urban Employee Basic Medical Insurance (UEBMI) and the Urban Resident Basic Medical Insurance (URBMI) [9, 10]. Most ESKD patients in urban China were enrolled in one of these schemes, but these two schemes covered different sub-populations and designed their own financing structure [9]. Furthermore, the Chinese government enhanced insurance reimbursement for patients with major catastrophic diseases including ESKD in 2012, in order to reduce the OOP costs for these patients [8]. All basic medical insurance systems cover both HD and PD, but the reimbursement rates vary from 50 to 90% across regions with different socioeconomic statuses [8]. Therefore, assessing the direct medical costs of ESKD is important for the future planning of health insurance policies.

Many countries conducted cost analysis according to different types of RRT, including dialysis and transplantation [11–17]. However, only two studies examined the direct medical costs of RRT in China [7, 18]. They did not estimate the per-person costs that controlled for patient age, gender and comorbidities, nor did they report the outpatient and inpatient utilizations, or separate the costs of KT in the first year and second year, which were substantially different in healthcare utilizations and expenditures. Finally, they did not compare the differences in direct medical costs and the OOP spending for dialysis patients between two different urban health insurance schemes.

This study aimed to investigate the annual direct medical costs per capita among ESKD patients by different types of RRT - HD; PD; KT (first year); KT (second year), and by two urban health insurance schemes, using claims data from the largest city in Southern China and examined the composition of medical costs and healthcare utilizations among ESKD patients.

## Methods

### Data source

Guangzhou is the capital of Guangdong Province, the largest and most developed city in Southern China. Guangzhou’s health insurance has covered the costs of RRT, including HD, PD and KT since 2001 for ESKD patients, which was much earlier than most cities in China [19]. Therefore, patients covered by Guangzhou’s UEBMI and URBMI schemes can afford RRT and may be less likely to forego RRT for economic reasons, closing the treatment gap in RRT among ESKD patients. The detailed reimbursement policies and benefit packages of the UEBMI and URBMI schemes for ESKD patients from Guangzhou city in 2013 were summarized in Table 1. Data in this study were obtained from the UEBMI and URBMI claims database of Guangzhou city for the years 2010 through 2013, which contains sociodemographic information, the utilization of hospital-based outpatient and inpatient services (not all patients have both inpatient and outpatient utilization), direct medical costs of outpatient and inpatient care based on actual payments to providers. The most common comorbidities including hypertension, diabetes, coronary heart disease were linked using personal identifiers with a chronic patient registry under the Outpatient Chronic Disease Program from these two insurance schemes. By 2013, 96.6% of the registered residents were enrolled in the two insurance programs in Guangzhou city [20]. This study was approved by the Institutional Review Board of School of Public Health, Sun Yat-Sen University (No. 201533).

**Table 1:** Comparison of UEBMI and URBMI policies for ESKD patients in Guangzhou city in 2013

	UEBMI			URBMI			
Inception year	2002			2008			
Eligible population	Urban employed			Urban non-employed			
	(Employees; Retirees)			(Children & full-time Students; Unemployed adults;			
				Elderly residents not covered by the UEBMI scheme)			
Sources of funding	The employers contribute 6% of the employee’s salary			Government subsidy (70%) and individual premium (30%)			
	whilst the employees contribute 2%;			CNY440 to CNY1800 per person per year for residents			
	Retirees are exempted from premium contribution			(including government subsidy)			
Accounts	Medical Savings Account (including employee			Social Risk-pooling Account (all funds) for inpatient			
	contributions and 30% of employer contributions)			care and critical (i.e. chronic or fatal diseases including			
	for outpatient care; Social Risk-pooling Account (70%			ESKD) outpatient care			
	of employer contributions) for inpatient care and						
	critical (i.e. chronic or fatal diseases including						
	ESKD) outpatient care						
	CRITICAL OUTPATIENT			CRITICAL OUTPATIENT			
Benefit packages of Social Risk-pooling Account	(HD, PD, KT immunosuppression)			(HD, PD, KT immunosuppression)			
	Employees	Primary hospitals	90%	Children & students		Primary hospitals	85%
		Secondary hospitals	85%			Secondary hospitals	75%
Reimbursement rate^a^ (Outpatient care)		Tertiary hospitals	80%			Tertiary hospitals	65%
	Retirees	Primary hospitals	93%	Unemployed adults and		Primary hospitals	85%
		Secondary hospitals	89.5%	Elderly residents		Secondary hospitals	70%
		Tertiary hospitals	86%			Tertiary hospitals	55%
Reimbursed ceiling (Outpatient care)	HD and PD: No monthly ceiling			HD and PD: No monthly ceiling			
	KT immunosuppression: CNY6000 monthly			KT immunosuppression: CNY5500–6000 monthly			
	INPATIENT			INPATIENT			
Deductible: (Inpatient care)	Employees	Primary hospitals	CNY400	Children & students		Primary hospitals	CNY120
		Secondary hospitals	CNY800			Secondary hospitals	CNY240
		Tertiary hospitals	CNY1600			Tertiary hospitals	CNY480
	Retirees	Primary hospitals	CNY280	Unemployed adults and		Primary hospitals	CNY280
		Secondary hospitals	CNY560	Elderly residents		Secondary hospitals	CNY560
		Tertiary hospitals	CNY1120			Tertiary hospitals	CNY1120
Reimbursement rate^a^ (Inpatient care)	Employees	Primary hospitals	90%	Children & students		Primary hospitals	85%
		Secondary hospitals	85%			Secondary hospitals	75%
		Tertiary hospitals	80%			Tertiary hospitals	65%
	Retirees	Primary hospitals	93%	Unemployed adults and		Primary hospitals	75%
		Secondary hospitals	89.5%	Elderly residents		Secondary hospitals	65%
		Tertiary hospitals	86%			Tertiary hospitals	55%
Reimbursed ceiling (Inpatient care)	Six times of local employees’ annual average wage			Six times of local household disposable income			
	CNY382,512			CNY228,324			

Notes: Policy information was obtained from Statistical Bulletin of Guangzhou Social Insurance Bureau, and policy documents

ESKD patients were exempt from deductibles for HD, PD, KT immunosuppression in the outpatient sector in Guangzhou

^a^The percentages were the reimbursement rates of the eligible medical expenses that could be reimbursed from the Social Risk-pooling Account in Guangzhou

Abbreviations: *UEBMI* Urban Employee-based Basic Medical Insurance scheme, *URBMI* Urban Resident-based Basic Medical Insurance scheme, *ESKD* end-stage kidney disease, *HD* Haemodialysis, *PD* Peritoneal Dialysis, *KT* Kidney Transplantation, CNY Chinese Yuan

### Study design and patient selection

**Fig. 1:**
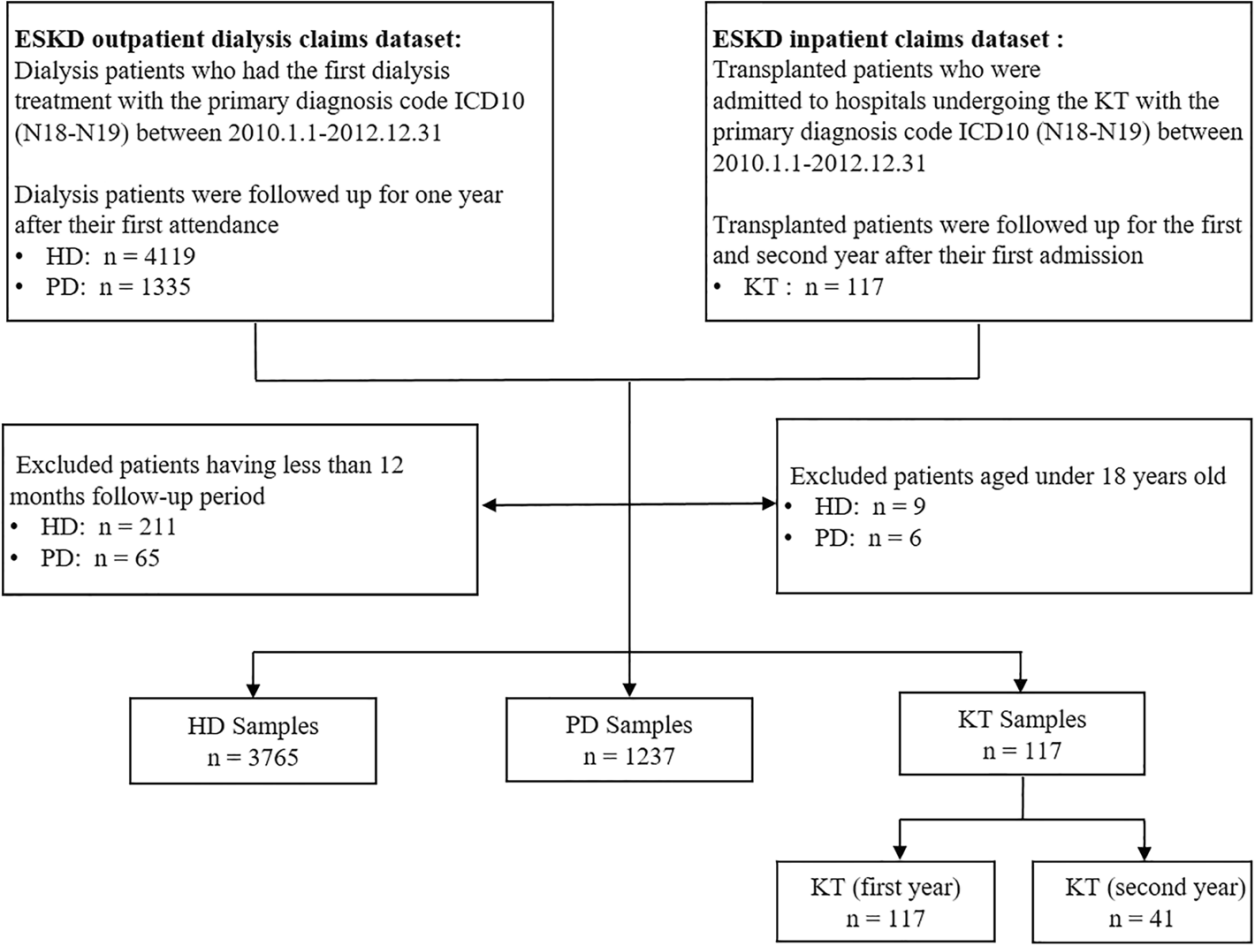
Sample selection framework Abbreviations: ESKD, End-stage kidney disease; HD, Haemodialysis; PD, Peritoneal Dialysis; KT, Kidney Transplantation

This was an observational cohort study designed to estimate the cost of ESKD according to different treatment strategies: HD, PD, KT (first year) and KT (second year). Patients admitted to hospitals in Guangzhou city with a primary diagnosis of ESKD were all included. We selected all the reimbursement claims submitted for outpatient and inpatient care between January 2010 and December 2012 using the International Classification of Diseases Tenth version (ICD-10) (N18-N19), and then followed up for one year (See Fig. 1).

The dialysis cohort was identified using the ESKD outpatient dialysis claims dataset that included all insured patients with ESKD receiving dialysis treatment in the outpatient sector of hospitals. Based on the types of dialysis, HD patients and PD patients who had the first dialysis treatment with the primary diagnosis code of ICD 10 (N18-N19) between January 1, 2010 and December 31, 2012 were identified and then followed up for one year after their first attendance. Not all dialysis patients received both outpatient and inpatient services during the follow-up period. For those patients who did have hospitalizations, they were linked using personal identifiers from the ESKD inpatient claims dataset to include their inpatient care information. All selected dialysis patients were also linked with a chronic patient registry using personal identifiers under the Outpatient Chronic Disease Program to include their information on three common comorbidities (hypertension, diabetes, coronary heart disease). For the patients not shown in the registry, we assumed that they had no comorbidities. We excluded patients who had a follow-up period that was less than 12 months and those under 18 years of age. Then we identified 3765 HD patients and 1237 PD patients.

The transplantation cohort was identified using the ESKD inpatient claims dataset that included patients undergone transplantation in the inpatient sector of hospitals. Transplanted patients who were admitted to hospitals undergoing the KT with the primary diagnosis code ICD 10 (N18-N19) between January 1, 2010 and December 31, 2012 were selected. This KT cohort included 117 patients, and they were followed up for the first and second year after their first admission, because different period would lead to obviously different costs for the patients with KT. These transplanted patients were then linked using personal identifiers from outpatient immunosuppression claims dataset to include their outpatient care information. All selected transplanted patients were also linked with a chronic patient registry using personal identifiers to get information on the aforementioned three common comorbidities. This study divided the observation period of the KT cohort into two parts, one was from the KT initiation up to the first year, the other was from the first year to the second year. Thus, 41 patients out of 117 KT (first year) patients were identified as KT (second year).

The final sample included 3765 HD, 1237 PD, 117 KT (first year), and 41 KT (second year) patients.

### Outcome measures

The primary outcome was the annual per patient medical costs incurred in the hospital outpatient and inpatient sectors. Secondary outcomes were the annual outpatient visits and inpatient admissions, length of stay per admission. Costs were adjusted using the Consumer Price Index (CPI) of 2013 in Guangzhou city [20], and were reported in Chinese Yuan (CNY) (US$1.0 = CNY6.2897 in 2013). The annual medical costs were not subject to discounting in this study. To compare the costs of different countries in different study period, we derived 2013 US dollar value by using consumer price indices of study countries in the years of costs and purchasing power parity (PPP) exchange rate in 2013 from the Organization for Economic Co-operation and Development (OECD) [21]. As suggested in Karopadi et al. [22] and Just et al. [23] ‘s studies, the cost ratio of HD versus PD (the annual per patient cost of HD divided by the annual per patient cost of PD) was reported in order to compare the relative costs of HD versus PD across different countries. For example, the cost ratio of 1.50 for HD versus PD means that HD treatment is on average 50% more expensive than PD [22].

### Cost estimation

The claim databases contain information on direct medical costs of outpatients and inpatients with different types of RRT from healthcare system’s perspective, including the total amount paid by the insurers and the patients. The total direct medical costs were separated into laboratory and diagnostic costs, non-medication treatment costs, medication costs, bed fees and costs of other services, including special caring fees, air-conditioning fees, based on the classification of costs used in the UEBMI and URBMI schemes. Laboratory and diagnostic costs included the expenses of physical tests and biochemical examinations. Medication costs were divided into costs for prescribed traditional Chinese Medicine and western medicine. Non-medication treatment costs were the expenditures of any other treatments except for medication, which consisted of surgical expenses, costs of anesthesia, blood transfusion expenses, and spending for medical consumables.

Costs of HD and PD were annual healthcare costs incurred in the outpatient and inpatient sectors, including costs for routine dialysis treatment and hospitalizations if needed. Because the first-year costs of dialysis and second year costs are expected to be similar, only the first-year costs of dialysis cohort were considered in this study. Around 16.9% HD patients (*N* = 699) and 13.8% PD patients (*N* = 184) did not have the complete one-year follow-up observations due to migration, death or dropping out of the program that we were not able to confirm in the claims dataset. In our cost estimation, we assumed that these patients should have continued to receive the same dialysis treatment (HD and PD) in the following months and had similar expenditures in each month. To estimate the annual costs of these HD and PD patients, we calculated the average monthly expenditures based on their observation months in the claims data and then multiplied that by twelve. We did not drop these patients because it may potentially lead to selection bias, but we conducted sensitivity analysis to assess the extent to which these patients with incomplete observations have on the estimated costs (described below in the statistical analysis).

In addition, costs of KT (first year) and KT (second year) were estimated separately, because the initial KT costs and maintaining KT costs were substantially different [11, 13, 14]. The costs from the first KT initiation up to the first year including the costs of kidney transplant procedure in the inpatient sector and the anti-immune treatment in the outpatient sector were estimated as the KT (first year) costs, while the costs from the first year up to the second year were considered as KT (second year) costs. The costs associated with the transplantation procedure were only incurred in the first year of KT but were not included in the second year.

The annual medical costs of different treatment strategies (HD, PD, KT in the first year, KT in the second year) were possibly influenced by several confounding factors. The covariates included in this study were age, gender, insurance types and presence of three common comorbidities. Age was categorized into four groups: 18–45 years old, 45–60, 60–75, 75 and older. Gender was dichotomized as male vs female, and insurance type was dichotomized as UEBMI vs URBMI. Comorbidities were measured as binary variables for the following conditions – whether having a hypertension, diabetes, or coronary heart disease. The rationale for choosing these confounders was based on the Andersen’s behavioral model [24]. In this conceptual framework, individual factors were chosen according to: (1) predisposing characteristics – existing conditions that predispose people to use or not use health services (for example age and gender); (2) enabling characteristics – conditions which facilitate or impede health services utilization including coverage of health insurance; and (3) need characteristics – conditions which health professionals recognize as requiring long-term health care treatment such as the presence of common comorbidities [24].

Information on patient characteristics (age, gender, type of insurance), hospital levels (primary, secondary, tertiary), outpatient services utilization (outpatient visits) and inpatient services utilization (inpatient admissions, length of stay (LOS) per admissions, readmission in 15 days) was also obtained from the claims database. The number of outpatient visits was reported by the number of outpatient claims except for HD patients. HD patients often received dialysis treatment in the outpatient setting three times per week, but they can only claim their reimbursement monthly based on insurance policy in Guangzhou. We assumed that the HD patients received three times of dialysis per week (12 visits per month). Thus, the annual number of HD outpatient visits was calculated by the number of outpatient claims multiplied by twelve.

### Statistical analysis

Descriptive statistics were used for demographic information and healthcare utilization measures. Continuous variables were presented as mean **±** standard deviation (SD) or median (25th–75th), and categorical variables as frequency (percentage). The independent two-sample T-test was used to compare the statistical differences in outpatient visits, inpatient care admissions and LOS for the HD and PD patients. The two-proportion Z-test was used to determine whether the differences between the proportions of patients having hospitalization for the HD and PD patients were statistically significant. In order to compare the costs for HD and PD patients by insurance types, the independent two-sample T-test was used to investigate whether the differences in medical costs between the two health insurance schemes within the HD subgroup and PD subgroup were statistically significant. The percentage of OOP spending between the two health insurance schemes was analysed using two-proportion Z-test to determine if significant differences were present. Given the number of statistical tests being conducted, a Bonferroni adjustment to the false positive rate was applied to the study, and the adjusted alpha level for statistical significance was 0.0083 (alpha = 0.05/6). The 99.17% CIs were presented around the effect estimates to reflect the adjusted alpha level (1–0.0083 = 0.9917). When comparing costs across HD, PD, KT (first year) and KT (second year) patients, annual medical costs were estimated after adjusting for patient age, gender, insurance types and presence of three comorbidities, using the generalized linear models (GLM). Advantages of the GLM approach are that predictions are made on the raw cost scale, so that no retransformation is required, and that they allow for heteroskedasticity through the choice of distributional family [25]. Based on the results from the link test and modified Park test for the choice of appropriate link function and family [25], log link function with gamma distribution was selected in this study. Bootstrapping method [26] with 1000 replicates was used to derive standard errors and bias-corrected 95% CI.

We conducted two sensitivity analyses. First, we estimated the adjusted costs of PD and HD by dropping out those patients from the analytic sample who did not have complete observations during the one-year follow-up period and compared the confidence intervals of these new estimates with those original ones. The more the confidence intervals overlapped, the less sensitive the model was to the selection process. Second, we tested the assumption on the number of outpatient visits among the HD patients by assuming they used dialysis for twice or once a week, instead of three times a week, in order to assess if this assumption would substantially influence the estimates and the conclusion. All statistical analyses were performed using Stata version 12.0 (Stata Corporation, College Station, TX, USA).

## Results

### Patient characteristics

Patients in the HD group (*n* = 3765) were on average aged 57.5 years, while patients in the PD group (*n* = 1237) were younger (51.1 years), and patients in the KT group (first year) (*n* = 117) were the youngest (37.0 years) among all groups (Table 2). All groups were predominantly male, especially among the transplanted patients, in which more than 70% were men. Regarding the comorbidities, 54.2% of the HD patients, 37.8% of the PD patients, 32.5% of the KT (first year) patients and 29.3% of the KT (second year) patients had hypertension. Most of the patients - 88.7% in the HD group and 91.9% in the PD group - were under the UEBMI scheme. All patients in the KT group were under the UEBMI scheme.

**Table 2:** Baseline patients characteristics, n (%) or mean ± standard deviation (SD) or median (25th–75th)

	HD	PD	KT (first year)	KT (second year)
No. Patients	3765	1237	117	41
Gender: Male	2089(55.5)	683(55.2)	82(70.1)	31(75.6)
Age (years)				
Mean ± SD	57.5 ± 15.8	51.1 ± 16.1	37.0 ± 9.7	39.0 ± 11.2
Age Group				
18–45 years	827(22.0)	472(38.2)	93(79.5)	29(70.7)
45–60 years	1166(31.0)	357(28.9)	23(19.7)	11(26.8)
60–75 years	1168(31.0)	305(24.7)	1(0.9)	1(2.4)
≥ 75 years	604(16.0)	103(8.3)	0(0.0)	0(0.0)
Comorbidities				
None	1520(40.4)	703(56.8)	77(65.8)	28(68.3)
Hypertension	2042(54.2)	468(37.8)	38(32.5)	12(29.3)
Diabetes	1057(28.1)	271(21.9)	7(6.0)	4(9.8)
Coronary	362(9.6)	52(4.2)	2(1.7)	0(0.0)
Insurance type				
UEBMI	3341(88.7)	1137(91.9)	117(100.0)	41(100.0)
URBMI	424(11.3)	100(8.1)	0(0.0)	0(0.0)

*HD* Haemodialysis, *PD* Peritoneal Dialysis, *KT* Kidney Transplantation, *UEBMI* Urban Employee-based Basic Medical Insurance scheme, *URBMI* Urban Resident-based Basic Medical Insurance scheme

### Healthcare utilization

#### Outpatient care

HD patients had on average 218.6 outpatient visits per year for dialysis treatment, and most of them underwent treatment in tertiary hospitals (78.9%) (Table 3). The corresponding number of outpatient visits per year for PD patients was 12.6 visits yearly, and the majority of patients received treatment in tertiary hospitals (96.5%). As for transplanted patients, they visited outpatient sector 21.2 times in the first year and 16.2 times in the second year. Mean annual outpatient visits for HD patients was significantly higher than those for PD patients (HD versus PD: 218.6 visits versus 12.6 visits; difference = 205.9 visits, 99.17% confidence intervals (CI) = 194.1 to 217.6 visits, *P* = 0.000). We also tested the assumption on the number of outpatient visits among the HD patients by assuming they used dialysis for twice or once a week. Compared with the number of PD outpatient visits (12.6 visits) reported, regardless of the assumption on number of outpatient visits per month, HD patients had significantly larger volume of outpatient visits than PD patients. The assumption would not influence the main conclusion.

**Table 3:** Annual outpatient and inpatient care utilization, % or mean ± standard deviation

	HD	PD	Difference (99.17%CI)	*P* value	KT (first year)	KT(second year)	Difference (99.17%CI)	*P* value
No. Patients	3765	1237			117	41		
Outpatient services								
Annual outpatient visits	218.6 ± 22.7	12.6 ± 5.3	205.9 (194.1–217.6)	0.000^a^	21.2 ± 9.8	16.2 ± 4.0	5.0 (2.0–7.9)	0.000^a^
Hospital level: Primary (%)	0.5	0.0	\	\	0.0	0.0	\	\
Hospital level: Secondary (%)	20.6	3.5	5.2 (3.5–7.8)	0.000^b^	0.0	0.0	\	\
Hospital level: Tertiary (%)	78.9	96.5	28.7 (26.9–30.6)	0.000^b^	100.0	100.0	\	\
Inpatient services								
Patients having hospitalization (%)	26.8	23.3	24.7 (23.1–26.4)	0.000^b^	100.0	\	\	\
Annual inpatient admissions	2.0 ± 1.9	1.6 ± 0.9	0.4 (0.2–0.6)	0.000^a^	3.4 ± 4.5	\	\	\
Length of stay per admission (days)	12.0 ± 10.2	14.2 ± 11.5	−2.2 (−4.3 - -0.3)	0.003^a^	28.1 ± 14.0	\	\	\
Readmission in 15 days (%)	3.9	3.5	0.6 (−2.8–3.9)	0.642^b^	5.1	\	\	\
Hospital level: Primary (%)	0.4	0.0	\		0.0	\	\	\
Hospital level: Secondary (%)	21.3	5.6	6.9 (3.5–13.0)	0.000^b^	0.0	\	\	\
Hospital level: Tertiary (%)	78.3	94.4	25.6 (22.2–29.3)	0.000^b^	100.0	\	\	\

*HD* Haemodialysis, *PD* Peritoneal Dialysis, *KT* Kidney Transplantation

^a^*p*-values were based on the independent two-sample T-test; ^b^p-values were based on the two-proportion Z-test. A Bonferroni adjustment was applied: the adjusted alpha level was 0.0083 (alpha = 0.05/6) and 99.17% Confidence Intervals (CI) were presented

#### Inpatient care

There were 26.8% HD patients and 23.3% PD patients had hospitalizations during the follow-up period, and the proportion of PD patients was significantly higher (*P* = 0.000). Mean annual inpatient admissions for patients on HD was significantly higher than those for patients on PD (HD versus PD: 2.0 visits versus 1.6 visits, difference = 0.4 visits, 99.17% CI = 0.2 to 0.6 visits, P = 0.000), but the LOS per admission was significantly shorter in the HD group than the PD group (HD versus PD: 12.0 days versus 14.2 days, difference = − 2.2 days, 99.17% CI = − 4.3 to − 0.3 days, *P* = 0.003). Most of the HD and PD patients received inpatient services from tertiary hospitals (78.3 and 94.4%). The transplanted patients only had hospitalizations in the first year and had 3.4 inpatient admissions and 28.1 days for LOS per admission.

#### Cost composition

**Fig. 2:**
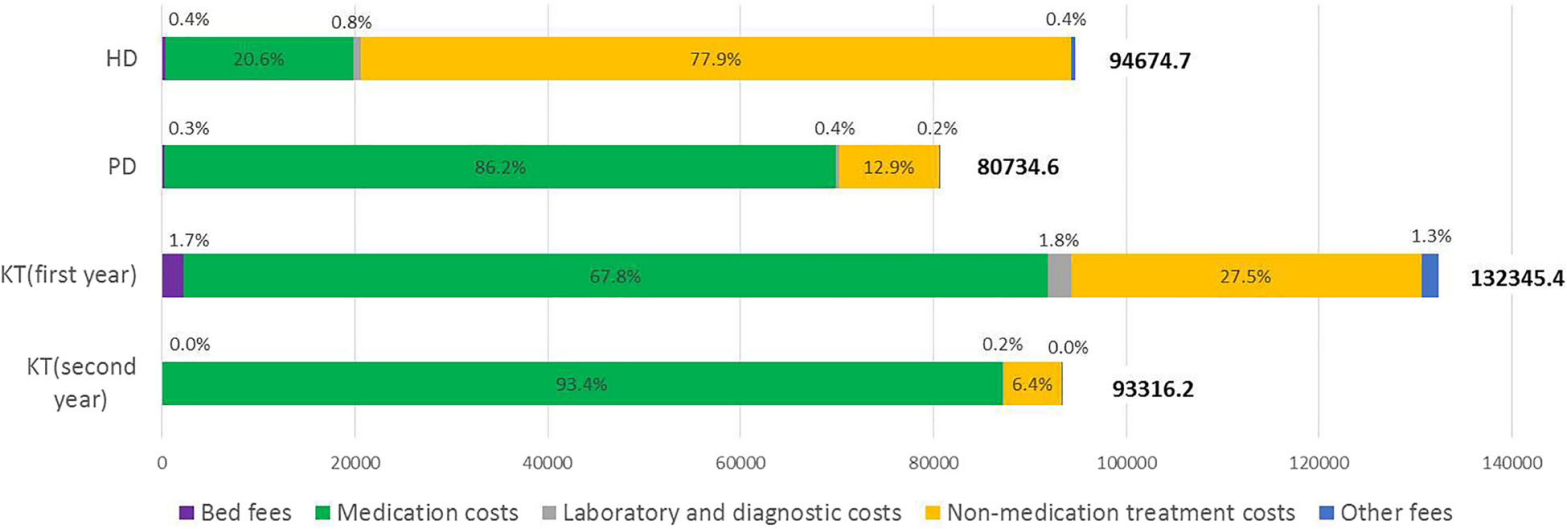
Composition of annual medical costs by types of renal replacement therapies Notes: All costs were based on Chinese Yuan (CNY). Abbreviations: HD, Haemodialysis; PD, Peritoneal Dialysis; KT, Kidney Transplantation

The mean annual costs for patients on HD (CNY94,674.7; US$15,052.3) was significantly higher than the mean annual costs for patients on PD (CNY80,734.6; US$12,836.0) (difference = CNY13,940.1, 99.17% CI = CNY10,825.5 to CNY17,054.7, *P* = 0.000) (Table 4). The non-medication treatment costs for patients on HD occupied the biggest proportion of total medical costs (77.9%), but the largest cost component in the PD group was medication costs related to fluids (86.2%) (see Fig. 2). The highest mean annual cost was observed in the KT (first year) group (CNY132,345.4; US$21,041.6), and it was significantly higher than the mean annual cost of the KT (second year) group (CNY93,316.2; US$14,836.4) (difference = CNY39,029.2, 99.17% CI = CNY22,547.6 to CNY55,510.8, *P* = 0.000). Medication costs took up the largest proportion of the total medical costs for the transplanted patients in the first year (67.8%) and second year (93.4%).

**Table 4:** Unadjusted annual medical costs per patient by types of renal replacement therapies, in Chinese Yuan (CNY)

	Overall HD	Overall PD	Difference (99.17%CI)	*P* value	KT (first year)	KT (second year)	Difference (99.17%CI)	*P* value
Composition of total costs	3765	1237			117	41		
Total annual medical costs								
Mean	94,674.7	80,734.6	13,940.1 (10,825.5–17,054.7)	0.000^a^	132,345.4	93,316.2	39,029.2 (22,547.6–55,510.8)	0.000^a^
SD	45,034.2	32,459.0			39,385.0	31,350.6		
Laboratory and diagnostic costs								
Percentage of total cost (%)	0.8	0.4			1.8	0.2		
Mean	730.1	307.7	422.4 (258.7–586.2)	0.000^a^	2324.9	187.3	2137.6 (1730.8–2544.5)	0.000^a^
SD	2870.4	1432.1			1337.5	567.2		
Non-medication treatment costs								
Percentage of total cost (%)	77.9	12.9			27.5	6.4		
Mean	73,747.8	10,377.7	63,370.1 (61,632.0–65,108.1)	0.000^a^	36,421.5	5996.1	30,425.4 (27,096.7–33,754.1)	0.000^a^
SD	27,286.9	17,060.3			12,483.6	2969.6		
Medication costs								
Percentage of total cost (%)	20.6	86.2			67.8	93.4		
Mean	19,498.0	69,633.0	−50,135.1 (−52,198.9 - -48,071.3)	0.000^a^	89,668.5	87,130.6	2537.9 (−12,788.2–17,863.9)	0.654^a^
SD	18,190.4	25,409.8			32,098.5	30,766.0		
Bed Fees								
Percentage of total cost (%)	0.4	0.3			1.7	0.0		
Mean	349.4	265.6	83.8 (−1.3–168.9)	0.009^a^	2235.5	0.0	2235.5 (1619.5–2851.4)	0.000^a^
SD	1406.7	795.6			2480.8	0.0		
Other fees								
Percentage of total cost (%)	0.4	0.2			1.3	0.0		
Mean	354.1	150.6	203.5 (116.7–290.4)	0.000^a^	1695.0	2.2	1692.8 (1519.3–1866.3)	0.000^a^
SD	1166.2	943.2			698.5	13.2		
Out-of-pocket spending								
Percentage of total cost (%)	13.0	14.3	−1.1 (−2.0 - -0.3)	0.000^b^	23.7	16.8	6.9 (3.2–10.8)	0.000^b^
Mean	12,323.9	11,516.7	807.2 (−49.2–1663.5)	0.013^a^	31,378.6	15,703.6	15,675 (9026.7–22,323.3)	0.000^a^
SD	11,461.9	9318.3			17,453.4	11,971.4		

*HD* Haemodialysis, *PD* Peritoneal Dialysis, *KT* Kidney Transplantation

UEBMI, Urban Employee-based Basic Medical Insurance scheme; URBMI, Urban Resident-based Basic Medical Insurance scheme;

^a^*p*-values were based on the independent two-sample T-test; ^b^p-values were based on the two-proportion Z-test. A Bonferroni adjustment was applied: the adjusted alpha level was 0.0083 (alpha = 0.05/6) and 99.17% Confidence Intervals (CI) were presented

Regarding the direct medical costs by types of insurance, the mean annual medical costs for the HD patients under the UEBMI scheme (CNY96,746.0; US$15,381.7) was significantly higher than HD patients under the URBMI scheme (CNY78,353.3; US$12,457.4) (difference = CNY18,392.6, 99.17% CI = CNY13,383.9 to CNY23,401.4, *P* = 0.000) (Table 5). Within the PD subgroup, the mean annual medical costs for the PD patients under the UEBMI scheme (CNY81,879.4; US$13,018.0) was significantly higher than PD patients under the URBMI scheme (CNY67,718.1; US$10,766.5) (difference = CNY14,161.3, 99.17% CI = CNY7,276.4 to CNY21,046.1, *P* = 0.000). However, the percentage of OOP expenses out of the total costs for the HD patients under the UEBMI scheme (10.7%) was significantly lower than that for patients with the URBMI on HD (35.4%) (P = 0.000). Regarding the PD patients, the proportion of OOP spending out of the total costs for the patients with the UEBMI (12.6%) was significantly lower than that for patients with the URBMI on PD (37.4%) (P = 0.000), demonstrating that these two insurance schemes had different benefit packages as mentioned above.

**Table 5:** Direct annual medical costs per patient by insurance types, in Chinese Yuan (CNY)

	HD				PD			
	UEBMI HD	URBMI HD	Difference (99.17%CI)	*P* value	UEBMI PD	URBMI PD	Difference (99.17%CI)	*P* value
Composition of total costs	3341	424			1137	100		
Total annual medical costs								
Mean	96,746.0	78,353.3	18,392.6 (13,383.9–23,401.4)	0.000^a^	81,879.4	67,718.1	14,161.3 (7276.4–21,046.1)	0.000^a^
SD	45,704.5	35,379.5			32,876.2	23,776.1		
Laboratory and diagnostic costs								
Percentage of total cost (%)	0.8	0.7			0.4	0.5		
Mean	750.5	569.6	180.9 (−72.4–434.2)	0.059^a^	307.7	307.3	0.4 (− 209.5–210.3)	0.996^a^
SD	2988.8	1658.9			1481.3	653.5		
Non-medication treatment costs								
Percentage of total cost (%)	77.8	78.9			12.4	19.3		
Mean	75,259.5	61,835.7	13,423.8 (10,170.0–16,677.6)	0.000^a^	10,138.7	13,094.6	− 2955.9 (− 7852.2–1940.4)	0.108^a^
SD	27,382.7	23,338.7			17,006.4	17,519.7		
Medication costs								
Percentage of total cost (%)	20.7	19.6			86.7	79.7		
Mean	20,023.9	15,353.7	4670.2 (2697.8–6642.5)	0.000^a^	71,013.1	53,941.5	17,071.6 (9905.2–24,238.1)	0.000^a^
SD	18,606.7	13,831.7			24,932.4	25,639.5		
Bed Fees								
Percentage of total cost (%)	0.4	0.4			0.3	0.4		
Mean	354.1	312.5	41.6 (− 129.0–212.30)	0.518^a^	267.0	250.2	16.8 (−147.8–181.3)	0.785^a^
SD	1428.2	1224.8			813.0	564.9		
Other fees								
Percentage of total cost (%)	0.4	0.4			0.2	0.2		
Mean	363.3	281.8	81.5 (−8.6–171.5)	0.020^a^	152.8	124.5	28.3 (−74.5–131.1)	0.466^a^
SD	1222.2	549.8			980.9	256.2		
Out-of-pocket spending								
Percentage of total cost (%)	10.7	35.4	−24.5 (−25.8 - -23.0)	0.000^b^	12.6	37.4	25.0 (−27.7 - -22.3)	0.000^b^
Mean	10,371.1	27,711.6	−17,340.5 (−19,655.8 - -15,025.2)	0.000^a^	10,303.4	25,311.9	−15,008.5 (− 18,084.7 - -11,932.2)	0.000^a^
SD	8627.2	17,716.8			8083.5	11,186.0		

*HD* Haemodialysis, *PD* Peritoneal Dialysis, *KT* Kidney Transplantation

UEBMI, Urban Employee-based Basic Medical Insurance scheme; URBMI, Urban Resident-based Basic Medical Insurance scheme

^a^*p*-values were based on the independent two-sample T-test; ^b^p-values were based on the two-proportion Z-test. A Bonferroni adjustment was applied: the adjusted alpha level was 0.0083 (alpha = 0.05/6) and 99.17% Confidence Intervals (CI) were presented

#### Adjusted annual costs

After adjusting for age, gender, insurances types, and three comorbidities, the annual medical costs of HD patients were estimated to be CNY94,760.5 (US$15,066.0; 95%CI: CNY85,166.6–106,972.2); while those of PD patients were estimated to be CNY80,762.9 (US$12,840.5; 95%CI: CNY76,249.8–85,498.9) (Table 6). The adjusted annual cost ratio of HD versus PD was 1.17 (95% CI: 1.12–1.25).

**Table 6:** Adjusted annual medical costs^a^ per patient by types of renal replacement therapies (CNY, 95%CI)

	Overall HD	Overall PD	KT (first year)	KT (second year)
No. Patients	3765	1237	117	41
Total annual medical costs (CNY)	94,760.5(85,166.6–106,972.2)	80,762.9(76,249.8–85,498.9)	132,253.0(114,009.9–153,858.6)	93,155.3(61,120.6–101,989.1)
Laboratory and diagnostic costs (CNY)	724.7(257.2–1500.2)	309.1(195.7–481.8)	2325.4(2017.4–3260.2)	/
Non-medication treatment costs (CNY)	73,801.2(69,423.2–79,717.3)	10,390.0(8323.3–13,617.0)	36,404.8(33,090.6–48,643.0)	6013.0(5194.4–7458.4)
Medication costs (CNY)	19,527.1(14,657.2–26,696.9)	69,654.7(63,253.9–76,369.8)	89,598.9(69,504.8–106,162.1)	86,958.0(53,530.9–95,595.2)
Bed fees (CNY)	350.1(133.9–743.3)	266.4(177.1–407.8)	2226.3(1648.0–4363.7)	/
Other fees (CNY)	356.3(157.6–668.3)	151.0(85.8–252.1)	1698.6(1575.1–2179.4)	3.1(0.6–9.2)
Out-of-pocket spending (CNY)	12,302.0(9820.6–15,682.1)	11,508.3(7759.0–14,559.9)	31,281.3(24,042.6–36,188.1)	15,670.5(10,387.4–19,025.4)

*HD* Haemodialysis, *PD* Peritoneal Dialysis, *KT* Kidney Transplantation

^a^Adjusted for age, gender, insurance types and three comorbidities (hypertension, diabetes, coronary) status using the generalized linear models;

CNY, Chinese Yuan; CI, Confidence Interval

We conducted sensitivity analyses and estimated the new adjusted costs of HD and PD and new CIs by dropping those patients who did not have complete observations during the one-year follow-up period. The new adjusted costs of HD patients were CNY89,995.2 (95%CI: CNY83833.6–101,825.6); while the new adjusted costs of PD patients were CNY78,226.2 (95%CI: CNY74248.5–83,407.6). We found that these new 95% CIs from the sensitivity analyses and our original 95% CIs cost range reported above indeed overlapped, which suggested that this sample selection process did not significantly affect the adjusted cost estimates (Additional file 1: Table S1).

ESKD patients who received KT incurred higher (CNY132,253.0, US$21,026.9; 95%CI: CNY114,009.9–153,858.6;) costs in the year of initiation and lower annual costs (CNY 93,155.3, US$14,810.8; 95%CI: CNY61,120.6–101,989.1) in the second year, mainly attributing to the costs of admission for the transplant operation.

## Discussion

This was a retrospective cohort study conducted with a large ESKD sample in Guangzhou city, Southern China. We found the estimated per-person annual medical costs for patients on HD were CNY94,760.5 (US$15,066.0), higher than those for patients on PD (CNY80,762.9; US$12,840.5). The estimated annual cost ratio of HD versus PD was 1.17 (95% CI: 1.12–1.25). The estimated per-person annual medical costs of KT in the first year were CNY132,253.0 (US$21,026.9), and in the second year were CNY93,155.3 (US$14,810.8). This was the first study using sample from the claims database of an entire city to examine the direct medical costs of ESKD patients by four different types of RRT - HD, PD, KT (first year), KT (second year) - and compare health care costs under two different urban insurance schemes in China.

Comparing the findings of this research to those previous studies conducted in other countries [12, 13, 16, 27], a great difference in the estimated methods and results was found. Our cost estimates were much lower than those in the United States (US$87,638 for HD and US$73,612 for PD in 2014; US$86,221 for HD and US$72,422 for PD in 2013 after PPP adjustment) [27], and in Korea (€34,554 for HD and €25,806 for PD in 2013; US$49,566 for HD and US$37,017 for PD in 2013 after PPP adjustment) [13]. The differences in estimated costs might be attributable to the varied health care systems and structures across countries. For instance, the health care professional fees in China were set at a very low rate by the Chinese government [28], compared to those in the aforementioned countries. In particular, the fee schedule for HD treatment was much higher than that for PD treatment, while many services provided by doctors and nurses to PD patients such as patient training and follow-ups were not charged in China [29]. However, the cost ratio of HD versus PD (1.17) was similar to that in other countries. A comprehensive review reported that the cost of HD was between 1.03 and 2.35 times the cost of PD in 10 out of 14 Asia and Middle East countries [22]. In this study, the direct medical cost among HD patients was mainly driven by the non-medication treatment costs (77.9%), including the costs of staff salaries (physicians, nurses, technicians, auxiliaries), dialysis equipment, arteriovenous fistulas, specific dialysis-related services (dialysers, liners) [11]. Consistent with previous studies [12], the largest cost contributor in the PD group was medication costs related to dialysis fluids (86.2%), and 90% of PD solutions in China are imported [8]. The variation in direct medical costs between HD and PD patients might be accounted for by the higher hospitalization costs in the HD group, since there were more patients utilizing inpatient services among the HD patients, consistent with an Italy-based study [15].

Among the transplanted patients, the estimated annual medical cost of KT (first year) per capita (US$21,026.9) in 2013 were found to be much higher than that of KT (second year)(US$14,810.8), and this was consistent with previous studies in other countries [11, 13, 14, 17]. The yearly medical costs for KT (first year) and KT (second year) were US$23,393 and US$10,028 in Turkey in 2001 (US$29,866 for KT first year and US$12,803 for KT second year in 2013 after PPP adjustment) [11]. The noticeably higher medical costs for the KT in the first year were due to organ evaluation costs, transplant admission hospital costs including operation, hospital readmission, immunosuppression, physician and follow-up charges [17].

Our estimated annual medical costs per patient for the HD and the PD groups were similar to those in the two China-based studies. Sun et al. [7] presented that the per capita medical expenses were CNY104,700(2014) for HD and CNY92,300(2014) for PD in Nanjing city, while Neil et al. [18] reported they were CNY98,204 and CNY84,141. However, none of those studies estimated the adjusted costs that controlled for patient age, gender, insurance types and comorbidities. The number of annual outpatient visits and inpatient admissions for the HD group was higher than that for the PD group, which was first reported in cost-related studies in China, consistent with the findings in a Sweden-based study [12]. Although the number of transplanted patients was limited, this study was the first reported the direct medical costs of KT (first year) and KT (second year) in China. Different from the situation in other countries [30], fewer live donors are available for KT, since the Chinese government has required all hospitals to stop using organs from executed prisoners, and the civilian organ donation is the sole source for organ transplant in China [31]. Among different RRT modalities, KT would save costs in the long run [32], but organ shortage remains a challenge in China [31]. The alternative is dialysis – either HD or PD. Previous study has demonstrated that PD is a less expensive therapy than HD where the benefits are driven by cost savings of PD over HD [33]. In this study the annual per patient medical cost of PD patients was lower than that for HD patients, which was consistent with previous studies. The high prevalence of ESKD coupled with limited medical and economic resources highlights the need for strategies to maximize the use of PD in China [34]. To reduce the financial burden of China’s health insurance funds, the Chinese government should consider increasing PD penetration rates and reducing hospitalization costs. In addition, strategies such as pre-ESKD management program for patients with chronic kidney disease adopted in the UK [35] and Taiwan [36], could potentially delay disease progression and reduce high economic burden faced by patients and the healthcare system.

This study also analyzed the differences in direct medical costs for dialysis patients between two urban health insurance schemes while the two previous China-based studies did not cover. ESKD was one of the major catastrophic diseases covered by the health insurance with a high reimbursement rate in China, so that more patients with ESKD can obtain RRT [34]. The direct medical costs for the HD and PD patients with the UEBMI coverage were higher than those with the URBMI coverage, but the former had a lower rate of OOP spending. This result might be explained by the following reasons. The UEBMI and URBMI schemes covered different subpopulations with sources of funding and reimbursement policies set up differently, causing disparities in health care expenditures and utilizations [37]. HD and PD patients who were covered by the UEBMI scheme had higher rates of reimbursement and more comprehensive services coverage [10], which may induce higher annual medical expenses. On the other hand, patients covered by the URBMI scheme had limited services coverage [10], which might discourage them from consuming expensive services [38]. We suggested that to reduce the OOP expenditures, the two urban medical insurance schemes should be further consolidated to narrow the disparities in benefit packages across the programs [9, 10].

This study had several limitations. First, direct medical costs in dialysis patients who were not followed the complete year were annualized to get their complete 12-month costs, thus we might overestimate the annual costs of these patients. However, our sensitivity analysis showed that this had no statistically significant impact on our adjusted cost estimates. Second, we assumed the HD patients received three times dialysis per week to calculate their outpatient care visits, which may overestimate the number of HD outpatient visits. But our sensitivity analysis suggested that this would not influence the main conclusion. Third, when we estimated the adjusted annual per patient medical costs, some other confounding factors such as clinical severity factors (eg. urine volume, haemoglobin, serum albumin), education level, occupation and economic level were not included in this study because such data were not available in the claims data, thus our estimates could have been improved if these variables were measured. Fourth, the study population was limited to urban enrollees under two insurance schemes in one city of China, which cannot represent the whole Chinese population and could limit generalizability. Fifth, by linking the outpatient claims dataset, inpatient claims dataset and chronic patient registry under the Outpatient Chronic Disease Program with personal identifiers, some patient information may not be complete. Future studies should consider adding more survey-based information. Finally, the information on three comorbidities (hypertension, diabetes, coronary heart disease) was obtained from the chronic patient registry, so we likely miss information on other comorbidities who did not participate in this program.

## Conclusions

The direct medical costs of ESKD patients were high and different by types of RRT and insurance in China. PD patients has the lowest adjusted annual medical costs among four different types of RRT (HD, PD, KT in the first year, KT in the second year), suggesting that using more PD when appropriate might lower the economic burden of the insurance program. The high prevalence of ESKD coupled with limited economic resources highlights the need for strategies to maximize the use of PD in China. The findings can be used to conduct cost-effectiveness research on different types of RRT for ESKD patients that provides economic evidence for health policy design in China. The direct medical costs for the HD and PD patients under the UEBMI scheme were higher than those patients under the URBMI scheme. Such information can also be used by policy decision makers in urban insurance programs evaluation and health resources allocation.

## Supplementary information

**Additional file 1: Table S1.** Sensitivity Analyses New Adjusted annual medical costs* per patient by types of dialysis (CNY, 95%CI).

## Data Availability

The datasets used and analyzed during the current study are available from the corresponding author on reasonable request.

## Abbreviations

CI: Confidence interval
CNY: Chinese Yuan
CPI: Consumer price index
ESKD: End-stage kidney disease
GLM: Generalized linear model
HD: Haemodialysis
ICD-10: International Classification of Diseases Tenth version
KT: Kidney transplantation
LOS: Length of stay
OECD: Organization for Economic Co-operation and Development
OOP: Out-of-pocket
PD: Peritoneal dialysis
PPP: Purchasing power parity
PSM: Propensity score matching
RRT: Renal replacement therapy
SD: Standard deviation
UEBMI: Urban Employee-based Basic Medical Insurance
URBMI: Urban Resident-based Basic Medical Insurance
