# Supplementary material for: Direct medical costs of end-stage kidney disease and renal replacement therapy: a cohort study in Guangzhou City, southern China
Source: BMC Health Serv Res. 2020 Feb 14;20:122. doi: 10.1186/s12913-020-4960-x (PMC7023821; doi:10.1186/s12913-020-4960-x)
Supplement: Supplementary file 1 — Additional file 1: Table S1. Sensitivity Analyses New Adjusted annual medical costs* per patient by types of dialysis (CNY, 95%CI). [file 12913_2020_4960_MOESM1_ESM.docx]

**Supplement File**

**Table S1: Sensitivity Analyses New Adjusted annual medical costs* per patient by types of dialysis (CNY, 95%CI)**

|  | Overall HD | Overall PD |
| --- | --- | --- |
| No. Patients | 3066 | 1053 |
| Total annual medical costs (CNY) | 89995.2(83833.6-101825.6) | 78226.2(74248.5-83407.6) |
| Laboratory and diagnostic costs (CNY) | 419.9(194.4-840.4) | 266.2(145.7-550.5) |
| Non-medication treatment costs (CNY) | 71507.1(67964.2-77247.1) | 9892.7(8216.1-13403.9) |
| Medication costs (CNY) | 17633.2(14455.5-24513.3) | 67734.1(61133.6-73997.4) |
| Bed fees (CNY) | 201.2(84.2-430.3) | 213.4(115.7-495.1) |
| Other fees (CNY) | 235.8(112.3-488.6) | 127.5(47.9-312.8) |
| Out-of-pocket spending (CNY) | 11231.1(8654.2-14880.6) | 11042.9(7263.1-13819.9) |

HD, Haemodialysis; PD, Peritoneal Dialysis; KT, Kidney Transplantation;

*The results of sensitivity analyses including the new adjusted costs of HD and PD and new CIs after dropping those patients who did not have complete observations during the one-year follow-up period. Adjusted for age, gender, insurance types and three comorbidities (hypertension, diabetes, coronary) status using the generalized linear models;

CNY, Chinese Yuan; CI, Confidence Interval
